# Perfluorotetradecanoic Acid (PFTeDA) Induces Mitochondrial Damage and Oxidative Stress in Zebrafish (*Danio rerio*) Embryos/Larvae

**DOI:** 10.3390/toxics10120776

**Published:** 2022-12-12

**Authors:** Neep Patel, Emma Ivantsova, Isaac Konig, Christopher L. Souders, Christopher J. Martyniuk

**Affiliations:** 1 Center for Environmental and Human Toxicology, Department of Physiological Sciences, College of Veterinary Medicine, University of Florida, Gainesville, FL 32611, USA; 2 Department of Chemistry, Federal University of Lavras (UFLA), Lavras 37200-900, Brazil; 3 UF Genetics Institute, Interdisciplinary Program in Biomedical Sciences, Neuroscience, University of Florida, Gainesville, FL 32611, USA

**Keywords:** perfluorinated chemicals, behavior, mitochondria, adverse outcome, mechanism of action

## Abstract

Industrial and consumer products, such as pesticides, lubricants, and cosmetics, can contain perfluorinated compounds (PFCs). Although many short-chain PFCs have been linked to physiological and behavioral changes in fish, there are limited data on longer-chain PFCs. The objective of this study was to determine the potential impact of perfluorotetradecanoic acid (PFTeDA) exposure on zebrafish (*Danio rerio*) during early developmental stages. We measured several endpoints including gene expression, mitochondrial bioenergetics, and locomotor activity in zebrafish. Survival, timing of hatching, and deformity frequency were unaffected by PFTeDA at the concentrations tested (0.01, 0.1, 1, and 10 µM) over a 7-day exposure period. The expression levels of mitochondrial-related genes (*cox1* and *mt-nd3*) and oxidative stress-related genes (*cat, hsp70,* and *hsp90a*) were increased in larval fish with exposure to 10 µM PFTeDA; however, there was no change in oxidative respiration of embryos (i.e., basal respiration and oligomycin-induced ATP-linked respiration). Reactive oxygen species were reduced in larvae treated with 10 µM PFTeDA, coinciding with the increased transcription of antioxidant defense genes. Both the visual motor response test and light–dark preference test were conducted on 7 dpf larvae and yielded no significant findings. This study improves current knowledge regarding toxicity mechanisms for longer-chain PFCs such as PFTeDA.

## 1. Introduction

Industrial and consumer products such as pesticides, lubricants, and cosmetics contain chemicals in the form of perfluorinated compounds. Perfluorinated compounds (PFCs) are molecules comprised of carbon chains saturated with fluorine groups. The chemical structure provides stability to PFCs, preventing biodegradation [1]. Extended use of industrial products has led to the release of PFCs into the natural environment on a global scale and chemicals such as perfluorooctanoic acid (PFOA), perfluorooctanesulfonate (PFOS), and perfluorononanoic acid (PFNA) are often detected in environmental samples (i.e., soil, air, and water). Perfluorinated compounds can persist in bodies of water, and bioaccumulation can occur in aquatic organisms such as fish, leading to the potential development of physiological and morphological deficiencies. For instance, perfluorinated compounds exert developmental toxicity onto fish embryos, inducing edemas and spinal malformations [2].

Perfluorinated compounds can be further defined by possessing carboxylic acids in the form of perfluorinated alkyl acids (PFAA). PFAAs vary based on the length of the carbon chain. Consequently, two main types of perfluorinated alkyl acids contribute to bioaccumulation: short-chain and long-chain PFAAs. The chain length of PFAAs has a greater contribution to the chemical’s toxicity compared to the type of functional group present [3].

Perfluorotetradecanoic acid (PFTeDA) is a long-chain PFAA consisting of a chain of 14 carbons. This molecule is found in industrial and commercial products including photographic films, firefighting foams, detergents, and insecticides [4]. Concentrations of PFTeDA have been reported across the globe and it has been detected in both aquatic environments and within organisms; however, most data are derived from wastewater sludge and tissues. Shellfish in several bodies of water within France have been reported to contain PFTeDA ranging from wet weights between 0.014 ng/g and 0.667 ng/g among 100% of samples collected [5]. In more heavily industrial-based regions in Asia, PFTeDA can reach a wet weight of up to 46 ng/g in wastewater sludge from treatment plants [6]. Boone et al. [7] measured 17 PFAS in treated and source water from 25 drinking water treatment plants across the US and found the lowest concentration minimum reporting level of PFTeDA to be 0.13 ng/L. PFTeDA was also detected in 80% of microplastics present in the Pearl River Delta in China at an average concentration of 0.67 ng/g of dry weight (dw), reaching a maximum of 11.3 ng/g dw [8]. As PFTeDA accumulates in the environment, humans can also become exposed to PFC. For instance, a study of 225 children from Taiwan revealed that children’s serum lipid arithmetic concentrations contained means of 30.7 ng/mL in boys and 27.4 ng/mL in girls of PFTeDA [9]. PFTeDA is thus detectable across a range of environmental samples, and studies addressing the potential toxicity of PFTeDA are thus warranted.

While toxicity data for PFTeDA are limited, other studies have been conducted on PFAAs that are closely related in structure. The closely related compound perfluorotridecanoic acid (PFTrDA) can negatively impact fish development. Exposure to PFTrDA can also lead to endocrine disruption in zebrafish (*Danio rerio*). This is noteworthy as the endocrine system regulates development and all biological systems, coordinating tissue patterning and physiology. Jo et al. [10] exposed zebrafish to concentrations of PFTrDA (0, 0.01, 0.1, 1, or 10 mg/L) for 120 days after fertilization and found a decrease in the production of testosterone in males at 0.01 mg/L. Other PFAAs also cause adverse effects in fish and PFAAs have been documented to affect gene expression [11], oxidative stress responses [12], and swimming behavior [13]. Taken together, there are broad mechanisms of action of PFAAs that can depend on their physiochemical properties such as chain length.

To characterize the potential toxicity of PFTeDA, we utilized zebrafish embryos and larvae due to their rapid generation time and development into free-swimming larvae. The objective of the research was to discern the developmental and behavioral effects of PFTeDA exposure on zebrafish larvae. Developmental effects were measured by recording deformities and survivorship each day during a continuous 7-day exposure. Bioenergetic effects were determined using an XFe24 Flux analyzer; gene expression analysis of mitochondrial-related and oxidative stress-related genes was conducted by real-time PCR. The Visual-Motor Response Test and the Light–Dark preference test were also employed in zebrafish to increase knowledge regarding the potential behavioral impacts of PFTeDA.

## 2. Materials and Methods

### 2.1. Chemicals

Perfluorotetradecanoic acid or PFTeDA (PESTANAL^®^, analytical standard; CAS Number: 376-06-7; EC Number: 206-803-4) was purchased from Millipore-Sigma (St. Louis, MO, United States). Oligomycin A (CAS no. 579-13-5, purity >99%), carbonyl cyanide 4-(trifluoromethoxy)phenylhydrazone (CAS no. 370-86-5, purity > 98%), rotenone (CAS no. 83-79-4, purity > 95%), and sodium azide (CAS no. 26628-22-8, ≥99.5%) were also purchased as analytical standards from Millipore-Sigma for mitochondrial assays. Solvent control dimethyl sulfoxide was also purchased from Millipore-Sigma (CAS Number: 67-68-5).

### 2.2. Embryos and Zebrafish Breeding

Embryos were obtained by breeding multiple sets of adult zebrafish (AB × Tu strain, 6–10 months of age) from a breeding colony housed at Animal Care Services, University of Florida. Zebrafish were maintained in tanks with an automated recirculating Pentair ecosystem; water leaving the tank went through a drum filter (catches large particulates ~30 microns), biofilter (catches bacteria), CO2 strippers, and UV light (disinfection) before returning to the tank. Adult zebrafish were reared in water with the following parameters: temperature of 27 ± 0.5 °C, pH of 7.2 ± 0.2, salinity of 0.70 ppt, saturated oxygen concentration of ~85% of air saturation, and a 14:10 h light/dark cycle. Zebrafish were fed a mixed diet of Gemma micro-powered formula and blood worms.

To produce embryos for this study, two females and two males were placed into a standard zebrafish breeding tank with plastic vegetation and a divider. The following morning at approximately 8 a.m., the divider was removed, and the fish were allowed to breed. Embryos were collected and maintained in the filtered system water, and multiple water changes were conducted to wash the embryos to lower the risk of contamination. Once in the laboratory, the embryos were transferred to embryo-rearing media [Stock embryo rearing media (ERM): 8 g NaCl, 0.4 g KCl, 0.358 g Na_2_HPO_4_, 0.72 g CaCl_2_, 0.6 g KH_2_PO_4_, 1.23 g MgSO_4_, 0.35 g NaHCO_3_, 1.92 L distilled water, pH~7.2)] [14]. All experimental procedures were approved by the Institutional Animal Care and Use Committee of the University of Florida (#201708562-01).

### 2.3. Experimental Design and Chemical Exposures

Chemical stock solutions were prepared by dilution in ERM with a pH value of 7.2 ± 0.1. The exposure solutions were made fresh daily and vortexed, and then used to replace the solution in the beakers. The exposure solutions were prepared by adding 6 µL of a stock solution (stored at −20 °C for the experiment in brown glass vials) into 6 mL of ERM. The EVOS™ FL Auto Imaging System (Thermo Scientific, Waltham, MA, USA) and a digital microscope were used to view the embryos. This imaging system was used to document the mortality, hatch rate, and deformities of the embryos. Before assigning embryos to a specific beaker, eggs were carefully examined to determine whether they were fertilized or unfertilized.

All experiments proceeded in the same fashion. For each experiment, zebrafish embryos were randomly assigned to 25 mL Pyrex beakers in groups of 20 fish per beaker containing 10 mL ERM. The glass beaker (n = 5/experimental group) was the experimental replicate. Exposure concentrations investigated in each assay are indicated in each subsequent section. Data for PFTeDA levels in the natural environment were scarce, so we selected a broad range [0.01 µM, 0.1 µM, 1 µM, and 10 µM] to test impacts on gene expression and behavior. We selected these concentrations as mitochondrial bioenergetics may be altered by PFTrDA at 0.1 to 1 µM concentrations [15], based upon an increased rate of state 4 oxygen consumption and evidence for disrupted membrane potential, indicating that PFTrDA is a strong uncoupler of oxidative respiration at these concentrations. Multiple exposure experiments were conducted with the same experimental design for 7 days. Seven-day-old larvae are robust for behavior assays and the timeframe is comparable to other early developmental chemical exposures in zebrafish [16,17]. All endpoints were measured in 7 dpf larvae, except for the mitochondrial bioenergetics assays, which could only be assessed in embryos. Embryos and larval fish were housed in a mini-incubator (Benchmark, MyTemp™ mini digital incubator) at a temperature of 27 ± 1 °C. Special care was taken to minimize contact of the embryos with the external environment by covering each beaker with cellophane with several small holes to prevent contamination. Dead embryos were removed from beakers daily and a 90% solution change with fresh chemical dilutions was conducted.

### 2.4. Mitochondrial Respiration Measurement

The XFe24 Extracellular Flux Analyzer (Agilent) was used to measure oxygen consumption rates (OCR) in zebrafish, following our protocols [18,19]. After plate calibration overnight, one embryo was selected from each of the Pyrex glass beakers (ERM, 0.1% DMSO, 1, 5, or 10 μM PFTeDA) and placed into an Islet Capture Microplate (N = 4 embryos/treatment). Concentrations of mitochondrial toxicants (oligomycin, FCCP, rotenone, and sodium azide) and cycling parameters of the assay have been reported previously [19]. The mitochondrial stress test was conducted at 27 ± 1 °C. OCR data were exported to PRISM (V9) using Wave Desktop 2.6 Software (Agilent Technologies, Santa Clara, CA, USA).

### 2.5. Reactive Oxygen Species

Methods for the ROS assay using 2ʹ,7ʹ-Dichlorofluorescin Diacetate (Calbiochem, Millipore Sigma, CAS 4091-99, or DCFDA) conducted in our laboratory were recently published (Ivantsova et al., 2022). In the current study, embryos were collected at 6 hpf and maintained in an incubator at 27 ± 1 °C. Media changes were conducted daily with new sterile ERM or renewed PFTeDA treatment for 7 days. Treatment groups included ERM, DMSO, 0.1, 1, and 10 µM PFTeDA. Larvae were then homogenized and processed for ROS using a Synergy™ H4 Hybrid Multi-Mode Microplate Reader. Total protein using a BCA assay (ThermoFisher Scientific, Waltham, MA, USA) was measured to express ROS as a normalized signal intensity/(µg/mL) protein.

### 2.6. Acridine Orange Staining

Acridine orange staining was conducted according to the methods outlined by others [20]. Briefly, zebrafish larvae were exposed to either ERM, DMSO, or one dose of 0.1, 1, or 10 µM PFTeDA for 7 days. Then, they were washed with ERM and stained with 2 μg/mL acridine orange solution (CAS 65-61-2, Sigma-Aldrich, St. Louis, MO, USA) for 30 min at ambient temperature in the absence of light. Five larvae from each biological replicate were used, totaling 25 larvae per treatment. After washing with ERM (five times for 30 s), apoptotic cells were visualized with an EVOS™ FL Auto Imaging System (ThermoFisher Scientific, USA) using a GFP filter at 10× magnification. Fluorescence patches of vivid green color denoted apoptotic cells. The fluorescence intensity was quantified using the histogram tool of the ImageJ software (U.S. National Institutes of Health, Bethesda, MD, USA; http://rsbweb.nih.gov/ij/) (accessed on 15 July 2022).

### 2.7. Quantitative Real-Time PCR

Following a 7-day exposure, larval fish were euthanized and flash-frozen in liquid nitrogen. Pools of larval fish were derived from individual beakers in the experiment and sample sizes were four to five beakers per treatment. The total number of fish used in each treatment was as follows: 0.1% DMSO (n = 101), 0.01 µM (n = 76), 0.1 µM (n = 94), 1 µM (n = 96), and 10 µM (n = 96) PFTeDA. The discrepancy in the beaker amounts was due to the samples for one of the beakers for the 0.01µM PFTeDA treatment being lost due to bursting from high pressures during flash freezing. Real-time PCR followed our established protocols using TRIzol^®^ Reagent (Life Technologies, Carlsbad, CA, USA) [21,22]. RNA integrity was evaluated using the RNA 6000 Nano Kit and 2100 Bioanalyzer (Agilent Technologies, Santa Clara, CA, USA). Total RNA was treated with TURBO DNA free™ Kit (ThermoFisher Scientific). The cDNA was synthesized using 1000 ng RNA (iScript™ cDNA Synthesis Kit, Bio-Rad, Hercules, CA, USA) as per the manufacturer’s instructions. Three “no reverse transcriptase (NRT)” controls were prepared in the same fashion, except water was used instead of the enzyme. The T100™ Thermal Cycler (BioRad, USA) was used to synthesize cDNA as per the manufacturer’s instructions.

Target genes measured in this study included those related to the mitochondria and oxidative stress (ATP synthase F0 subunit 6 (*atp06*), catalase (*cat*), cytochrome c1 (*cyc1*), MT-CO1 (mitochondrially encoded cytochrome c oxidase I) (*cox1*), cytochrome c oxidase IV (*cox-iv*), cytochrome c oxidase subunit 5a1 (*cox5a1*), heat shock protein 70 (*hsp70*), heat shock protein 90a (*hsp90a*), mitochondrially encoded NADH:ubiquinone oxidoreductase core subunit 1 (*mt-nd1*), mitochondrially encoded NADH:ubiquinone oxidoreductase core subunit 2 (*mt-nd2*), mitochondrially encoded NADH:ubiquinone oxidoreductase core subunit 3 (*mt-nd3*), superoxide dismutase 1 (*sod1*), superoxide dismutase 2 (*sod2*)). Primer sequences can be found in Supplemental Methods.

Transcripts used to normalize the expression data included ribosomal protein S18 and beta-actin (*rps18* and *bactin*). Real-time PCR (qPCR) was performed using the CFX Connect Real-Time PCR Detection System (BioRad) with SsoFast™ EvaGreen^®^ Supermix (BioRad, Hercules, CA, USA). The cDNA was diluted 1/20 before use and synthesis followed the cycles and primer concentrations recommended by BioRad. Normalized gene expression was extracted using CFX Manager™ software V3.0 using the relative ΔΔCq method (baseline subtracted) based on established methods [23]. Negative controls showed that DNase treatment sufficiently removed genomic DNA, and melt curve analysis revealed that each primer set yielded one amplicon. Furthermore, the expression data had three to five biological replicates per group. The reason for the variability in biological replicates reported for some genes was due to instances where a replicate did not amplify (i.e., flatlined) due to a PCR inhibitor or failed reaction. This was rare and occurred in one or two samples in total. Additionally, at the end of the qPCR analysis, we became limited in the amount of cDNA available, which prevented us from measuring select samples.

### 2.8. Visual Motor Response Test

The DanioVision ™ Observation was used to gather data on the behavioral characteristics of the zebrafish. Methods followed our established protocols for behavioral assessments [21,24]. Five separate experiments were conducted to assess the effects of PFTeDA on zebrafish locomotor activity after continuous exposure on day 7. Approximately four zebrafish larvae were selected from each experimental beaker (biological replicate) at random and placed into a 96-well plate for video recordings. On day 7, each run included the following numbers of fish per group: ERM (n = 16), 0.1% DMSO (n = 16), 0.01 µM (n = 16), 0.1 µM (n = 16), 1 µM (n = 16), and 10 µM (n = 16) PFTeDA. The 0.1% DMSO was the control group used for comparisons among groups and we observed that, at this concentration of DMSO, there was no effect on our larval fish assay [25,26,27].

### 2.9. Light–Dark Preference Test

The LDPT was used to assess the potential for PFTeDA to cause anti-anxiolytic or anxiolytic-like behaviors in larval fish. Details of the assay methodology are reported in our previous publication [26]. Other studies show that environmental chemicals can affect behaviors related to anxiety [28]. For the LDPT, a 96-well plate was utilized that had a left side that was dark and a right side that was not dark due to a cover placed over the bottom of the plate. Zebrafish larvae at 7 dpf were placed individually into a 96 square-well plate (Whatman, CAT#: WH7701-1651) containing 200 µL of media (n = 4 fish from each replicate beaker for a total of 16 fish per treatment). Three separate runs were conducted, and data were combined into one graph representing all runs.

### 2.10. Statistical Analysis

Statistical analysis and graphing were conducted with GraphPad V9.3 (La Jolla, CA, USA). Residual and homoscedasticity plots were used to assess normality and homogeneity of variances (Shapiro-Wilk test) before ANOVA. The survival data were assessed via a Log-rank (Mantel-Cox) test and hatch data were assessed through a two-way ANOVA with a Dunnet’s post-hoc correction test. Oxygen consumption rates over time were presented as mean ± S.D. and analyzed using ANOVA followed by a Tukey’s test. Differences among groups for the ROS and acridine orange staining were analyzed using ANOVA followed by a Tukey’s test. The expression levels of transcripts were analyzed using a one-way ANOVA followed by a Dunnett’s multiple comparisons test. All measured genes (*atp06, cat, cyc1, cox1, cox-iv, cox5a1, hsp70, hsp90a, mt-nd1, mt-nd2, mt-nd3, sod1,* and *sod2*) were log-transformed (Y = log(Y)) to conform to assumptions of normality and then used to generate graphs. Furthermore, groups with two replicates had the third replicate assigned the lowest detectable sample: these genes and groups were *cat* (1 sample), *cox4i1* (1 sample), and *hsp90a* (1 sample). In addition, we assessed the genes using simple linear regression analysis to determine whether there was a relationship between concentration and response (i.e., relative gene expression). The VMR and LDPT for locomotor activity were analyzed using a one-way ANOVA followed by a Holm-Šídák’s multiple comparisons test and a Dunnett’s multiple comparisons test. Distance traveled, frequency in the dark zone, mean time in the dark zone, and cumulative duration in the dark zone were presented as mean ± S.D. Means of experimental groups were compared to the mean value of the solvent control (DMSO) group. For all endpoints, the significance of the difference between groups was acknowledged at *p* < 0.05.

## 3. Results

### 3.1. Survival, Hatch Rate, and Morphological Deformities

**Figure 1:**
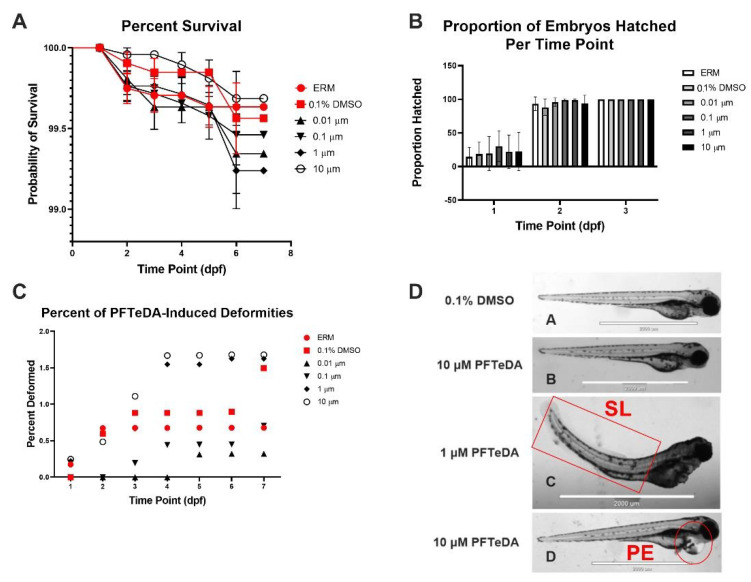
(**A**) Total percentage of surviving zebrafish embryos/larvae for ERM, 0.1% DMSO, 0.01 μM, 0.1 μM, 1 μM, and 10 μM PFTeDA over time based on Log-rank Mantel-Cox test. (**B**) Total percentage of hatched zebrafish embryos/larvae for ERM, 0.1% DMSO, 0.01 μM, 0.1 μM, 1 μM, and 10 μM PFTeDA over time. (**C**) Total percentage of deformities in zebrafish embryos/larvae for ERM, 0.1% DMSO, 0.01 μM, 0.1 μM, 1 μM, and 10 μM PFTeDA. (**D**) Representative zebrafish (**A**,**B**) and examples of spinal lordosis (SL) (**C**) and pericardium edema (PE) (**D**) at 3 dpf exposed to 0.1% DMSO, 1 μM, and 10 μM PFTeDA. The scale bar in each picture is 2000 µm.

Surviving zebrafish embryos/larvae were recorded daily over six separate experiments. Combined survival rates indicated that there was no significant difference between different concentrations of PFTeDA and the controls (Log-rank (Mantel-Cox) test, Chi-square = 4.47, d.f. = 5, *p* = 0.48). Each treatment and control group showed little to no mortality (<1%) throughout all the experiments up to 10 µM (Figure 1A). The hatch rate was also recorded via observation in the first three days to determine whether PFTeDA induced early or delayed hatching (Figure 1B). There were no significant differences between any of the treatment and control groups as all the zebrafish had hatched by 3 days post fertilization (two-way ANOVA with a Dunnett’s multiple comparisons test, F_(10, 50)_ = 0.30, *p* = 0.98). Embryos from the PFTeDA treatment groups hatched as expected, without significant differences in frequency in comparison to the control group (0.1% DMSO) (Figure 1B).

Overall, there was no trend of reoccurring malformations among the zebrafish exposed to differing concentrations of PFTeDA. Although deformities were noted in both the 1 and 10 μM PFTeDA treatment groups, the frequency of deformity overall was very low (<2%) (Figure 1C). Among the few deformities observed were pericardial edema (PE), yolk sac edema (YSE), spinal lordosis (SL), axial malformation (AM), and shortened trunk (ST) (Figure 1D).

### 3.2. Oxidative Consumption Rates

**Figure 2:**
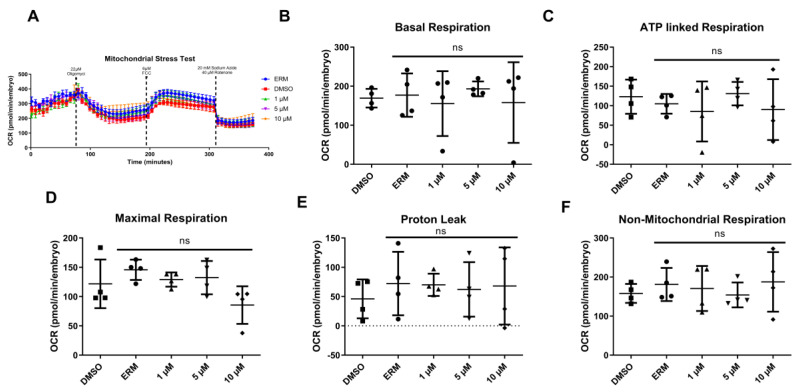
Oxygen consumption rate (OCR, pmol/min/embryo) for 30-h-old zebrafish embryos treated with PFTeDA for 24 h, beginning at 6 h. Effects of 1, 5, and 10 µM PFTeDA on mitochondrial endpoints compared to the vehicle control. (**A**) OCR over time, (**B**) Basal Respiration, (**C**) Oligomycin-induced ATP-Linked respiration, (**D**) FCCP-induced Maximal Respiration, (**E**) Proton Leak, and (**F**) Non-Mitochondrial Respiration. Each point is a biological replicate, and the horizontal line indicates the mean value of the group (mean ± S.D.) (one-way ANOVA with a Tukey’s multiple comparisons test, each group, n = 4 embryos from different exposure beakers). Symbols represent samples from different treatment groups. The ns = not significant.

Zebrafish embryos at ~6 hpf were treated with no chemical (ERM only), solvent control or 0.1% DMSO, or one concentration of either 1, 5, or 10 μM PFTeDA for 24 h (Figure 2A). There were no significant differences in the mean basal respiration (F_(4, 15)_ = 0.22, *p* = 0.92) (Figure 2B), oligomycin-induced ATP-linked respiration (F_(4, 15)_ = 0.52, *p* = 0.73) (Figure 2C), FCCP-induced maximum respiration (F_(4, 15)_ = 2.56, *p* = 0.082) (Figure 2D), proton leak (F_(4, 15)_ = 0.20, *p* = 0.93) (Figure 2E), or non-mitochondrial respiration (F_(4, 15)_ = 0.33, *p* = 0.86) (Figure 2F) between the control and PFTeDA treatments.

### 3.3. Reactive Oxygen Species

**Figure 3:**
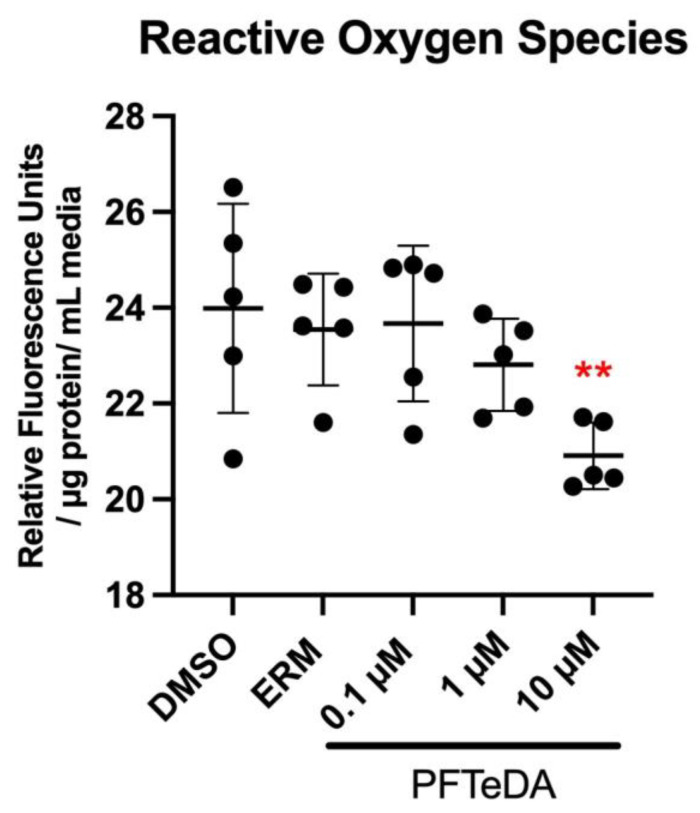
Normalized reactive oxygen species level in larval zebrafish treated with ERM, 0.1% DMSO, 0.1 μM, 1 μM, and 10 μM PFTeDA. Each point is a biological replicate and the horizontal line indicates the mean value of the group (mean ± S.D.) (one-way ANOVA followed by a Dunnett’s multiple comparisons test, n = 5/group). ** indicate that the values are significantly different from those of the control group at *p* < 0.01.

Zebrafish larvae were subjected to a 7-day continuous treatment with either 0.1, 1, or 10 µM PFTeDA. ROS levels were significantly different in larvae treated with PFTeDA (F_(4, 20)_ = 3.76, *p* = 0.020). Zebrafish exposed to 10 µM PFTeDA showed a decrease in ROS relative to all other groups (*p* = 0.0097) (Figure 3).

### 3.4. Acridine Orange

**Figure 4:**
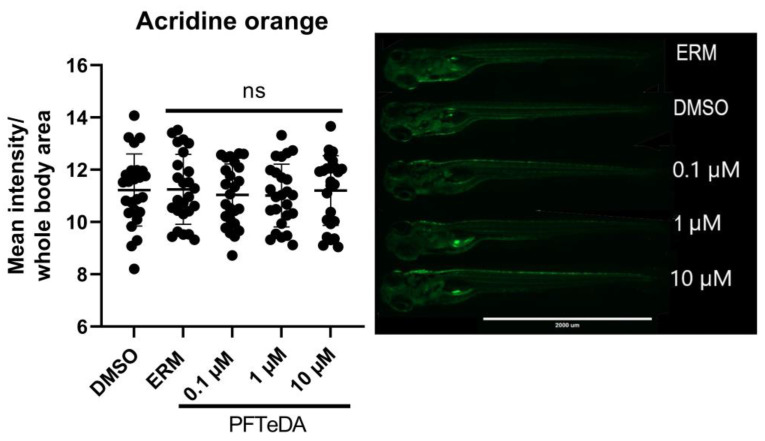
Acridine orange (apoptosis) staining in larval zebrafish treated with ERM, 0.1% DMSO, 0.1 μM, 1 μM, and 10 μM PFTeDA. Each point is a biological replicate and the horizontal line indicates the mean value of the group (mean ± S.D.) (one-way ANOVA followed by a Dunnett’s multiple comparisons test, n = 25/group).

The effect of PFTeDA exposure on zebrafish larvae apoptosis was assessed at 7 dpf. No significant differences were observed between the treatment and control groups (F_(4, 120)_ = 0.19, *p* = 0.95) (Figure 4).

### 3.5. Gene Expression Analysis

**Figure 5:**
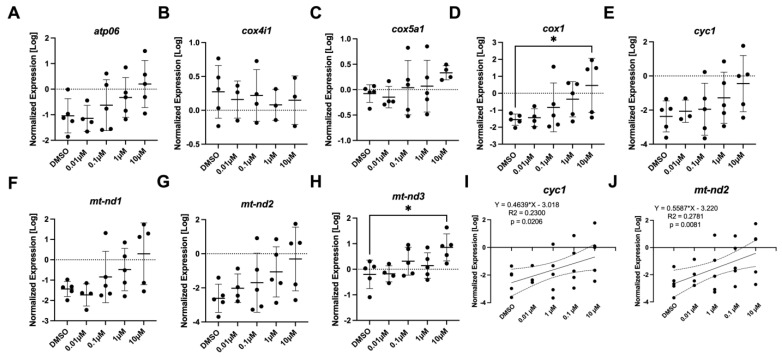
The expression levels of (**A**) *atp06*, (**B**) *cox4i1,* (**C**) *cox5a1,* (**D**) *cox1,* (**E**,**I**) *cyc1*, (**F**) *mt-nd1,* (**G**,**J**) *mt-nd2,* and (**H**) *mt-nd3* in 7-day old larval zebrafish exposed to either 0.1% DMSO, 0.01 μM, 0.1 μM, 1 μM, or 10 μM PFTeDA. Each point is a biological replicate, and the horizontal line indicates the mean value of the group (mean ± S.D.) (one-way ANOVA with a Dunnett’s multiple comparisons test, n = 3–5/group). An asterisk denotes a significant difference at * *p* < 0.05 from the solvent control.

**Figure 6:**
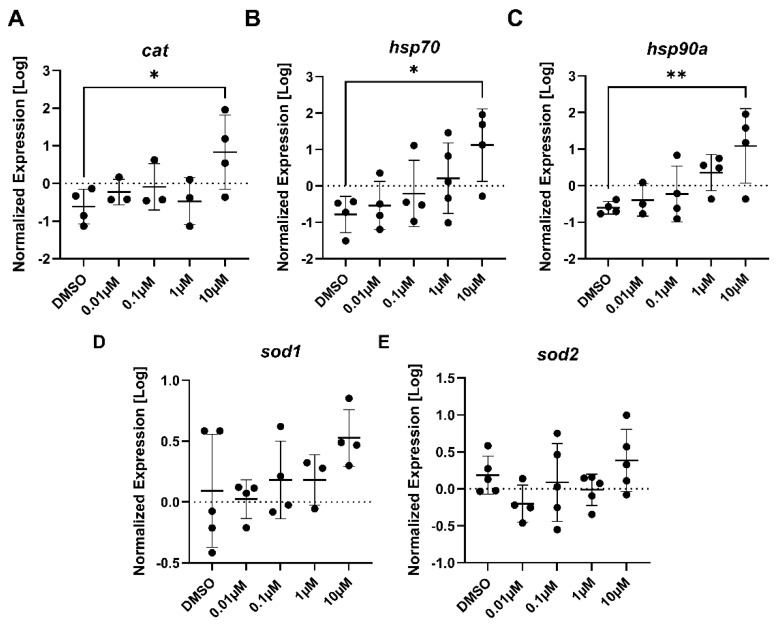
The expression levels of (**A**) *cat,* (**B**) *hsp70,* (**C**) *hsp90a,* (**D**) *sod1,* and (**E**) *sod2* in 7-day old larval zebrafish exposed to either 0.1% DMSO, 0.01 μM, 0.1 μM, 1 μM, or 10 μM PFTeDA. Each point is a biological replicate, and the horizontal line indicates the mean value of the group (mean ± S.D.) (one-way ANOVA with a Dunnett’s multiple comparisons test, n = 3–5/group). An asterisk denotes a significant difference at * *p* < 0.05 or ** *p* < 0.01 from the solvent control.

The effect of PFTeDA on the mRNA steady-state levels of transcripts associated with the mitochondria (Figure 5) and oxidative stress (Figure 6) was examined. The mitochondrial set of genes consisted of *atp06, cox4i1, cox5a1, cox1, cyc1, mt-nd1, mt-nd2,* and *mt-nd3*. Among the genes, *cox1* (Anova, F_(DFn, DFd)_, *p*-value; F _(4, 19)_ = 2.62, *p* = 0.068) and *mt-nd3* (F _(4, 19)_ = 3.56, *p* = 0.025) were altered in abundance, as determined by the post hoc Dunnett’s test (*p* = 0.036 and *p* = 0.013) between the 10 µM PFTeDA treatment and the control (0.1% DMSO), respectively (Figure 5D,H). Differences were detected between the two groups with a Dunnett’s test even when ANOVA results were *p* < 0.05, as individual tests have greater statistical power to detect differences among treatments. There were no changes in the expression levels of *atp06* (F _(4, 19)_ = 2.19, *p* = 0.11), *cyc1* (F _(4, 18)_ = 1.53, *p* = 0.23), *cox-iv* (F _(4, 13)_ = 0.17, *p* = 0.95), *cox5a1* (F _(4, 18)_ = 0.995, *p* = 0.436), *mt-nd1* (F _(4, 19)_ = 2.66, *p* = 0.065), or *mt-nd2* (F _(4, 19)_ =1.88, *p* = 0.157). However, it is worth noting that *cyc1* (Figure 5I) and *mt-nd2* (Figure 5J) showed a concentration-dependent response effect with coefficients of determination of 0.2300 (*p* = 0.0206) and 0.2781 (*p* = 0.0081), respectively.

The genes involved with stress consisted of *cat, hsp70, hsp90a, sod1,* and *sod2*. Each of the three genes, *cat* (F _(4, 12)_ = 2.82, *p* = 0.073), *hsp70* (F _(4, 16)_ = 3.21, *p* = 0.041), and *hsp90a* (F _(4, 14)_ = 4.21, *p* = 0.019) yielded a statistically significant difference (*p* = 0.032, *p* = 0.018, and *p* = 0.0092, respectively) in gene expression between the 10 µM PFTeDA treatment and the control (0.1% DMSO) (Figure 6A–C). Both *sod1* (F _(4, 15)_ = 1.54, *p* = 0.24) and *sod2* (F _(4, 19)_ = 1.68, *p* = 0.195) showed no changes in expression between the control and any treatment group for PFTeDA.

### 3.6. Locomotion Activity via the Visual Motor Response Test

The VMR test revealed that PFTeDA did not negatively impact larval activity at 0.01, 0.1, 1, or 10 µM, although, in each case, there was a difference in behavioral response due to the light–dark cycles [Trial 1 (F_(29, 270)_ = 44, *p* < 0.0001), Trial 2 (F_(29, 270)_ = 25, *p* < 0.0001), Trial 3 (F_(29, 270)_ = 21, *p* < 0.0001), Trial 4 (F_(29, 270)_ = 24, *p* < 0.0001), Trial 5 (F_(29, 270)_ = 13, *p* < 0.0001)] (Supplemental Figure S1).

### 3.7. Light–Dark Preference Test Analysis

**Figure 7:**
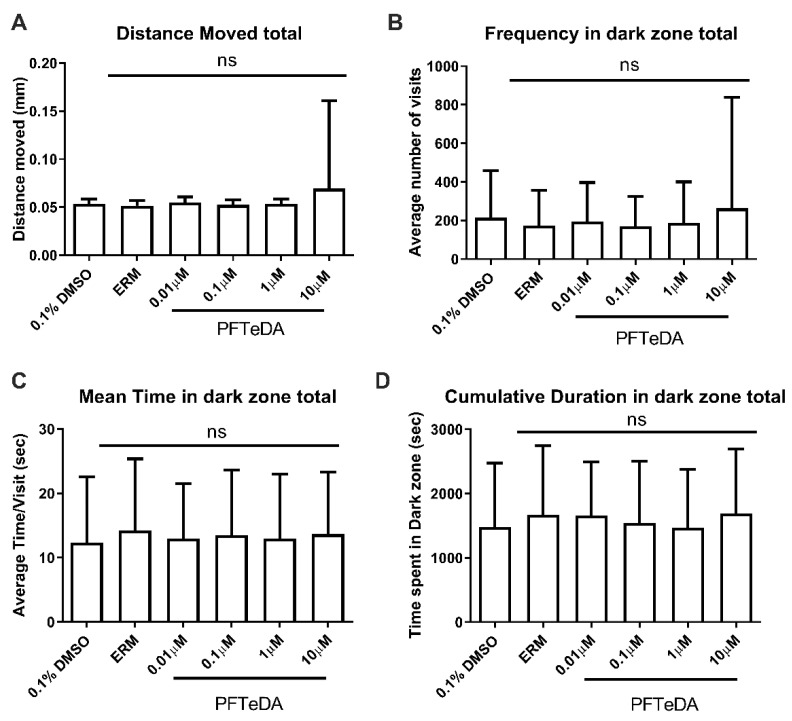
The light–dark preference test for anxiolytic behavior. (**A**) Total distance moved; (**B**) Frequency in dark zone; (**C**) Mean time in dark zone; (**D**) Cumulative duration in dark zone. Mean values are depicted by the individual columns that correspond to a treatment group (mean ± S.D.) (one-way ANOVA with a Dunnett’s multiple comparisons test, n = 31–46 fish/treatment, combined 3 trials). The ns = not significant.

We also hypothesized that PFTeDA may affect behaviors related to anxiety. Based on the light–dark preference test, PFTeDA did not alter the distance traveled in larval zebrafish (F_(5, 248)_ = 1.44, *p* = 0.21) (Figure 7). Additionally, PFTeDA did not significantly alter the frequency in the dark zone (F_(5, 248)_ = 0.57, *p* = 0.73), mean time in the dark zone (F_(5, 248)_ = 0.19, *p* = 0.96), or cumulative duration in the dark zone (F_(5, 248)_ = 0.46, *p* = 0.81).

## 4. Discussion

The chemical stability of perfluorinated compounds enables their persistence in the environment. Although the use of certain PFCs is restricted, these chemicals remain in the environment through runoff and pollute wildlife through bioaccumulation. Well-studied PFCs include both PFOA and PFOS, which are found in surface and groundwater globally and have been found to accumulate in human blood, proteins, and organs [29,30]. They are also notable for their toxicity to zebrafish development. Coupled with findings that indicate that carbon chain length may play a role in the toxicity of PFCs, it is important to investigate longer-chained PFAAs and their potential impact. With the use of zebrafish larvae, additional targets of toxicity can be investigated for PFTeDA-induced toxicity. Current information regarding the sub-lethal toxicity of PFTeDA in aquatic species is limited. However, similar-structured long-chain PFAAs have been investigated for their toxicity, such as perfluorodecanoic (PFDA), perfluoroundecanoic acid (PFUdA), and perfluorotridecanoic acid (PFTrDA). When appropriate, we compared the limited knowledge of PFTeDA toxicity to other PFAAs below.

The mortality and morphological changes in zebrafish were insignificant and suggested that PFTeDA has low toxicity to developing fish. The low mortality (<1%) coupled with the non-significant changes in hatch rate observed among the zebrafish exposed to each treatment group of PFTeDA resemble other results regarding similar long-chain PFAAs. In one study, zebrafish larvae were exposed to either PFTrDA or perfluoroundecanoic acid (PFUnDA) until 5 dpf was reached in concentrations of 0, 0.03, 0.1, and 0.3 mg/L [31]. This study also found that larval survival and time to hatch did not differ with exposure [31]. However, the study did show a decreasing trend in survival and hatchability with PFTrDA. In the current study, there was no distinguishable trend based on chemical concentration regarding either hatchability or survival with exposure to PFTeDA. In addition, PFTeDA did not result in any significant deformities as the frequency of deformity remained under 2%. In a study by Kim et al. [31], there was a significant increase in the percentage of deformities among zebrafish exposed to PFTrDA and PFUnDA. The most common deformity was YSE, which was reported in more than 20% of the zebrafish for treatments of 0.03 mg/L PFUnDA, 0.3 mg/L PFUnDA, 0.03 mg/L PFTrDA, and 0.1 mg/L PFTrDA. Out of the few deformities present in zebrafish exposed to PFTeDA, PE and SL were the most common, followed by YSE. It was also reported that both PFTrDA and PFUnDA had increased yolk sac size and decreased eyeball size [31]. Our investigation of PFTeDA did not assess the measurements of various zebrafish structures.

Mitochondrial dysfunction can lead to increased oxidative stress [32]. We observed a decrease in ROS levels in zebrafish larvae exposed to 10 µM PFTeDA. This suggests that exposure to high levels of PFTeDA may have induced overexpression of antioxidant enzymes, which was required to counteract the ROS being produced. In support of this, three transcripts (*cat*, *hsp70*, and *hsp90a*) were increased in zebrafish exposed to 10 µM PFTeDA. Catalase or *cat* is an antioxidant enzyme that combats oxidative stress. Furthermore, there was also a significant increase in *hsp70* and *hsp90a* transcripts that encode for heat shock proteins. Misfolded proteins are indicative of oxidative stress and require a defense mechanism such as heat shock protein induction [33,34]. Heat shock proteins serve as molecular chaperones that can resist oxidative stress; increased expression in zebrafish exposed to 10µM PFTeDA demonstrates that oxidative stress may be a toxicity mechanism for PFCs.

In addition, to combat the additional ROS produced through mitochondrial oxidative respiration, zebrafish upregulated several genes associated with Complex I and IV of the electron transport chain to maintain oxidative respiration. There was a significant increase in mitochondrial-related genes (e.g., *cox1, mt-nd2,* and *mt-nd3*). *Cox1* codes for cytochrome c oxidase subunit I, which is involved in Complex IV of the electron transport chain. *Mt-nd2* and *mt-nd3* code for NADH dehydrogenase 2 and NADH dehydrogenase 3, which are involved in Complex I of the electron transport chain [35,36]. These complexes are involved in shuttling electrons from electron carriers, with the end goal of reducing oxygen to drive chemiosmosis and ATP synthesis. We surmise that the upregulation of electron transport chain enzymes at the transcriptional level may be a compensatory mechanism to cope with the higher exposure concentrations of PFTeDA. One noteworthy observation was that basal respiration and oligomycin-induced ATP-linked respiration were not changed with exposure, indicating that the upregulation of the transcripts may facilitate normal oxygen consumption rates of mitochondria. Lastly, it is worth noting that there was no difference in apoptosis staining among the zebrafish exposed to PFTeDA, supporting the notion that anti-oxidant defense mechanisms may be able to resist any negative effects of PFTeDA.

Previous findings on PFCs and their impact on mitochondrial function and oxidative stress have varied in comparison to the effects observed here with PFTeDA. For instance, in a study where zebrafish were exposed to 0.1, 0.5, and 1 mg/L PFOA for 4 and 28 d, it was determined that there was a differential expression in genes related to the electron transport chain and oxidative phosphorylation [3]. The study reported an increase in mitochondrial membrane permeability that can dissipate the proton gradient necessary for ATP synthesis via chemiosmosis. The mechanism was hypothesized to be related to the PFC acting as a chemical agent that enhances the opening of mitochondrial permeability transition pores and reduces the ability to synthesize ATP [3]. Intriguingly, our study differed from these findings in that there was no significant effect of PFTeDA observed in relation to proton leak; perhaps these differences are attributable to the different chemical structures of PFAAs. Another study conducted by Liu et al. [37] investigated the production of ROS and changes to mRNA transcription due to PFCs via a 96 hpf exposure of PFNA at concentrations of 25, 50, and 100 µM. There were significant changes in the expression of mitochondrial-related genes: *atp06*, *cox1*, *ucp2* (uncoupling protein 2), and *mt-nd1*. In the study, *atp06* and *cox1* were downregulated in the 100 µM and 50 µM PFNA treatment groups, respectively. Here, PFTeDA at the highest concentration (10 µM) upregulated *cox1*, an opposite effect that was observed with PFNA [37]. All PFNA-treated zebrafish showed significant dose-dependent downregulation of *mt-nd1*; conversely, expression levels of *mt-nd2* and *mt-nd3* were increased in PFTeDA-treated larvae. The increase in expression levels of the mitochondrial anion carrier protein, uncoupling protein 2 (*ucp2*) [37], may also contribute to the increase in ROS generated among the 50 and 100 µM PFNA groups [37]. Additionally, *sod1* mRNA in PFNA-treated larvae was reduced, which may have resulted in an increase in ROS. Taken together, the study by Liu et al. (2015) is not consistent with our findings regarding PFTeDA (increased expression with reduced ROS) as the study reported down-regulation of mitochondrial- and oxidative stress-related genes with increases in ROS. However, Liu, Sheng, Zhang, and Dai [37] utilized concentrations that were much higher than those used in the current investigation with PFTeDA, and this may explain some of the discrepancies.

The absence of behavioral effects in both the Visual Motor Response Test and Light–Dark Preference Test among zebrafish exposed to PFTeDA suggests that the chemical does not influence behavioral changes. This finding regarding PFTeDA diverges from what was found by Ulhaq et al. [38], where there was a stronger change in locomotive behavior observed with PFC’s with longer chains and sulfonic groups. The study consisted of a 6-day zebrafish exposure to trifluoroacetic acid (TFAA), perfluorobutyric acid (PFBA), PFOA, PFNA, PFDA, perfluorobutane sulfonic acid (PFBS), and PFOS in concentrations ranging between 0.03 mg/L and 3000 mg/L [38]. Zebrafish exposed to the highest concentrations of TFAA, PFBS, PFOS, and PFNA were found to have a reduction in overall activity compared to the control group [38]. However, zebrafish exposed to PFBS and PFOS also had a higher active swimming speed alongside PFDA [38]. Although average swim speeds were higher among larvae exposed to PFBS and PFOS, there was an overall decrease in activity. Yet, in another study, zebrafish exposed to PFOA, PFNA, and PFOS for 14 days were noted to have increased swimming activity [13]. Furthermore, another study exposed 6 dpf zebrafish to PFOS for 1 h, resulting in a significant increase in swim speed among the two highest concentrations (4.0 and 8.0 µM PFOS) [39]. In another study that investigated the toxicity of functional groups among shorter-chain PFCs (Perfluorobutane sulfonate (PFBS), perfluoropentanoic acid (PFPeA), perfluorobutane sulfonamide (FBSA), and 4:2 fluorotelomer sulfonic acid (4:2 FTS)) at concentrations up to 100 µM, there were significant changes in larval activity after 120 hpf [40]. Hypoactivity in the dark phase of an LPR assay was reported in zebrafish exposed to FBSA at lower concentrations and in zebrafish exposed to an intermediate concentration of 4:2 FTS. Hyperactivity in the dark phase was observed among zebrafish exposed to higher concentrations of PFPeA, and no change in activity in the dark phase was observed with PFBS [40]. Although the study by Rericha et al. [40] was conducted on shorter-chained PFCs with different functional groups, there appears to be a similar trend with previous studies regarding how certain PFCs can either promote or inhibit activity among zebrafish larvae. The behavior of fish in response to PFCs appears to be variable in the literature as some studies report a reduction in activity, while others report hyperactivity. Our study with PFTeDA reveals no relationship between locomotor activity, light–dark preference, and PFTeDA exposure.

In summary, our investigation into the sub-lethal toxicity of PFTeDA reveals alterations of gene transcripts related to the mitochondria and oxidative stress. To potentially cope with the toxicity of PFTeDA, the expression levels of mitochondria-related genes were significantly upregulated, and this may have induced parallel changes in the upregulation of oxidative stress genes. No change in oxidative respiration was detected; however, the upregulation of genes that combat oxidative stress may have been sufficient to reduce ROS at the highest concentration of PFTeDA tested. In terms of activity and anxiolytic behavior, there was no evidence that PFTeDA induced behavioral impairments. Future studies should continue to examine longer-chained PFCs to discern chemical-specific effects in aquatic organisms.

## Data Availability

Not applicable.

## Supplementary Materials

The following supporting information can be downloaded at https://www.mdpi.com/article/10.3390/toxics10120776/s1, supplemental figures and tables. Supplemental Figure S1. The activity of 7-day zebrafish larvae exposed to ERM, 0.1% DMSO, or different concentrations of PFTeDA (0.01, 0.1, 1, or 10 µM). Supplemental Table S1. Primers used in this study [41,42,43,44,45,46,47,48].
