# Supplementary material for: Perfluorotetradecanoic Acid (PFTeDA) Induces Mitochondrial Damage and Oxidative Stress in Zebrafish (Danio rerio) Embryos/Larvae
Source: Toxics. 2022 Dec 12;10(12):776. doi: 10.3390/toxics10120776 (PMC9785682; doi:10.3390/toxics10120776)
Supplement: Supplementary file 1 [file toxics-10-00776-s001.zip › toxics-2038250-supplementary.pdf]

SUPPLEMENTAL DATA

Perfluorotetradecanoic acid (PFTeDA) Induces Mitochondrial Damage and Oxidative Stress in Zebrafish (*Danio rerio*) Embryos/Larvae

Neep Patel[1], Emma Ivantsova[1], Isaac Konig[2], Christopher L. Souders II[1], Christopher J. Martyniuk[1,3*]

[1]Center for Environmental and Human Toxicology, Department of Physiological Sciences, College of Veterinary Medicine, University of Florida, Gainesville, Florida, 32611, USA

[2]Department of Chemistry, Federal University of Lavras (UFLA), Lavras, Brazil 37200-900

[3]UF Genetics Institute, Interdisciplinary Program in Biomedical Sciences, Neuroscience

*Corresponding author: Christopher J. Martyniuk
Center for Environmental and Human Toxicology &
Department of Physiological Sciences
College of Veterinary Medicine, University of Florida
2187 Mowry Rd. Bldg 471 PO Box 110885
Gainesville, FL   32611
email: cmartyn@ufl.edu

Supplemental Table S1. Primers used in this study.

Supplemental Table 1. Primers used for real-time PCR analysis.				
Gene name	Gene Symbol	Forward (5' to 3')	Reverse (5' to 3')	Reference
ATP synthase F0 subunit 6	atp06	TTATCCTCGTTGCCATACTTC	AGTTGGTTTGTGAATCGTCC	Jin et al., 2010
beta-actin	bactin	CGAGCAGGAGATGGGAACC	CAACGGAAACGCTCATTGC	Wang et al. 2018
catalase	cat	CTCCTGATGTGGCCCGATAC	TCAGATGCCCGGCCATATTC	Sarkar et al., 2014
MT-CO1 (mitochondrially encoded cytochrome c oxidase I)	cox1	ACTTAGCCAACCAGGAGCAC	GGGTGGAAGAAGTCAGAAGC	Northam and LeMoine, 2019
cytochrome c oxidase subunit 5a	cox5a1	AAGCATAGATGTCTACGATTGTGAG	AGGCCAATTAAATAGAACACAAACAC	Duggan et al., (2011)
cytochrome c oxidase IV	cox-iv	CAAGTTTGTGCAGCAGCTG	CAAAGAAGAAGATTCCTGCAAC	Northam and LeMoine, 2019
cytochrome c1	cyc1	ACTTAGCCAACCAGGAGCAC	GGGTGGAAGAAGTCAGAAGC	McClelland et al. 2006
heat shock protein 70	hsp70	GAAGACGGCATCTTTGAGGTGA	GGGCCCTCTTGTTCTGACTGAT	Hahn et al., 2014
heat shock protein 90a	hsp90a	AGCTGGCGGATCGTTCACTGTC	AAAACTCGCCGTACTCCTCATTGG	Murtha and Keller et al., 2003
mitochondrially Encoded NADH:Ubiquinone Oxidoreductase Core Subunit 1	mt-nd1	AGCCATCTCAAGCCTAGCAG	ATTGTTTGCGCTACAGCTCG	AC024175.3
mitochondrially Encoded NADH:Ubiquinone Oxidoreductase Core Subunit 2	mt-nd2	GACCTACCAGCCACAGCTAC	TTGGGTCGTTTGTACCCGTC	AC024175.3

mitochondrially Encoded NADH:Ubiquinone Oxidoreductase Core Subunit 3	mt-nd3	ACCACTCCCATGAGGAGATCA	CTTGGGCTCATTCGTAGGCT	AC024175.3
ribosomal 18s	rps18	CGGAGGTTCGAAGACGATCA	TCGCTAGTTGGCATCGTTTATG	Wang et al. 2018
superoxide dismutase 1	sod1 (Cu/Zn SOD)	CAACACAAACGGCTGCATCA	TTTGCAACACCACTGGCATC	Sarkar et al., 2014
superoxide dismutase 2	sod2 (Mn SOD)	AGCGTGACTTTGGCTCATTT	ATGAGACCTGTGGTCCCTTG	Sarkar et al., 2014

Duggan AT, Kocha KM, Monk CT, Bremer K, Moyes CD. Coordination of cytochrome c oxidase gene expression in the remodelling of skeletal muscle. J Exp Biol. 2011 Jun 1;214(Pt 11):1880-7. doi: 10.1242/jeb.053322. PMID: 21562175.

Hahn, M.E., McArthur, A.G., Karchner, S.I., Franks, D.G., Jenny, M.J., Timme-Laragy, A.R., Stegeman, J.J., Woodin, B.R., Cipriano, M.J. and Linney, E., 2014. The transcriptional response to oxidative stress during vertebrate development: effects of tert-butylhydroquinone and 2, 3, 7, 8-tetrachlorodibenzo-p-dioxin. *PloS one*, *9*(11), p.e113158.

Jin, Y., Zhang, X., Shu, L., Chen, L., Sun, L., Qian, H., Liu, W. and Fu, Z., 2010. Oxidative stress response and gene expression with atrazine exposure in adult female zebrafish (Danio rerio). *Chemosphere*, *78*(7), pp.846-852.

McClelland, G.B., Craig, P.M., Dhekney, K. and Dipardo, S., 2006. Temperature-and exercise-induced gene expression and metabolic enzyme changes in skeletal muscle of adult zebrafish (Danio rerio). *The Journal of Physiology*, *577*(2), pp.739-751.

Murtha JM, Keller ET. Characterization of the heat shock response in mature zebrafish (Danio rerio). Exp Gerontol. 2003 Jun;38(6):683-91. doi: 10.1016/s0531-5565(03)00067-6. PMID: 12814804.

Northam, C. and LeMoine, C.M., 2019. Metabolic regulation by the PGC-1α and PGC-1β coactivators in larval zebrafish (Danio rerio). Comparative Biochemistry and Physiology Part A: Molecular & Integrative Physiology, 234, pp.60-67.

Sarkar, S., Mukherjee, S., Chattopadhyay, A. and Bhattacharya, S., 2014. Low dose of arsenic trioxide triggers oxidative stress in zebrafish brain: expression of antioxidant genes. Ecotoxicology and environmental safety, 107, pp.1-8.

Wang, X.H., Souders 2nd, C.L., Zhao, Y.H., Martyniuk, C.J. 2018. Paraquat affects mitochondrial bioenergetics, dopamine system expression, and locomotor activity in zebrafish (Danio rerio). Chemosphere. 191, 106-117.

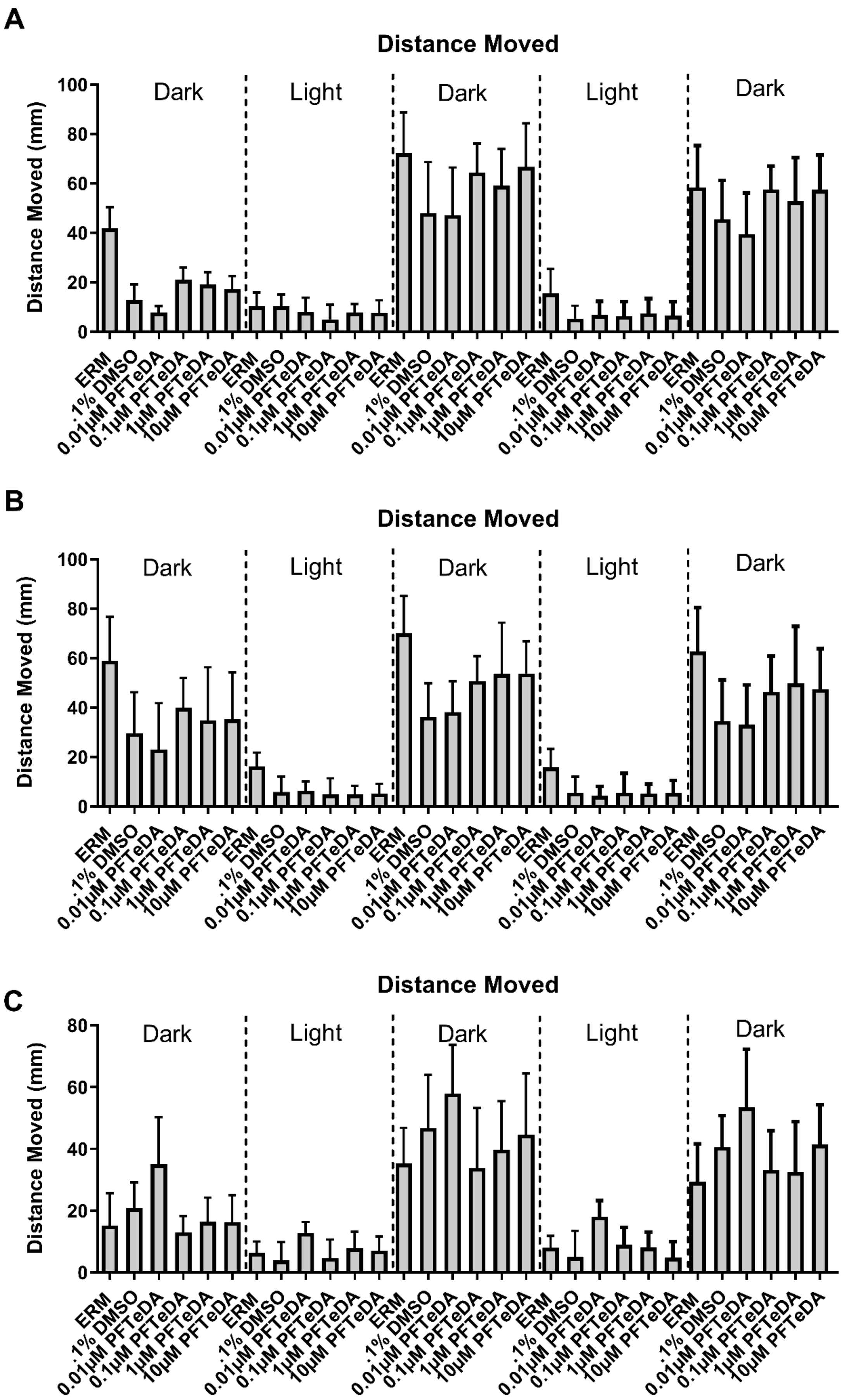
A
Distance Moved
Distance Moved (mm)
Dark
Light
Dark
Light
Dark
ERM
.1% DMSO
0.01µM PFTeDA
0.1µM PFTeDA
1µM PFTeDA
10µM PFTeDA
B
Distance Moved
Distance Moved (mm)
Dark
Light
Dark
Light
Dark
C
Distance Moved
Distance Moved (mm)
Dark
Light
Dark
Light
Dark

D

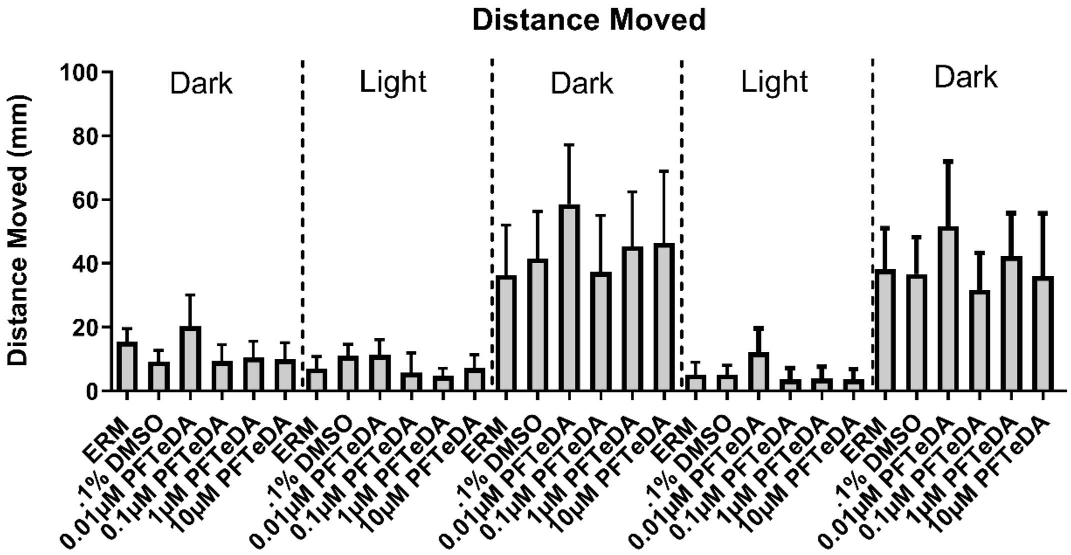

E

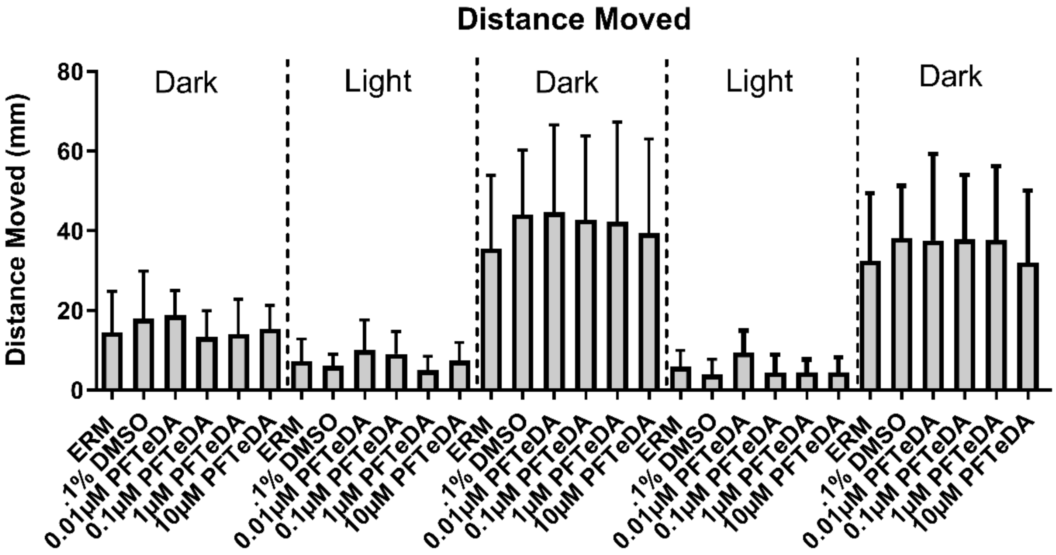

Supplemental Figure S1. The activity of 7-day zebrafish larvae exposed to ERM, 0.1% DMSO, or different concentrations of PFTeDA (0.01, 0.1, 1, or 10 µM). Mean values are depicted by the columns in each dark-light phase (mean ± S.D.) (One-Way ANOVA with a Holm-Šídák's multiple comparisons test, n=16/treatment/experiment).
